# Intussusception and COVID-19 in Children: A Systematic Review and Meta-Analysis

**DOI:** 10.3390/children9111745

**Published:** 2022-11-14

**Authors:** Saad Alhumaid, Zainab Al Alawi, Abdulrahman A. Alnaim, Mohammed A. Al Ghamdi, Muneera Alabdulqader, Khalid Al Noaim, Ali A. Rabaan, Koblan M. Al mutared, Nourah Al Dossary, Murtadha Alsuliman, Yameen Ali Almatawah, Ahmed Tawffeq AlOmran, Sarah Mahmoud Al HajjiMohammed, Duaa Riyadh Alfarhan, Hussain Ahmed Al Suwaiq, Manea M. Al mutarid, Mohammed Jamal Alkolib, Ranjan K. Mohapatra, Abbas Al Mutair

**Affiliations:** 1 Administration of Pharmaceutical Care, Al-Ahsa Health Cluster, Ministry of Health, Al-Ahsa 31982, Saudi Arabia; 2 Division of Allergy and Immunology, College of Medicine, King Faisal University, Al-Ahsa 31982, Saudi Arabia; 3 Department of Pediatric, College of Medicine, King Faisal University, Al-Ahsa 31982, Saudi Arabia; 4 Department of Paediatrics, King Fahad Hospital of the University, Imam Abdulrahman Bin Faisal University, College of Medicine, Dammam 34212, Saudi Arabia; 5 Pediatric Nephrology Specialty, Pediatric Department, Medical College, King Faisal University, Al-Ahsa 31982, Saudi Arabia; 6 Department of Pediatrics, College of Medicine, King Faisal University, Al-Ahsa 31982, Saudi Arabia; 7 Molecular Diagnostic Laboratory, Johns Hopkins Aramco Healthcare, Dhahran 31311, Saudi Arabia; 8 College of Medicine, Alfaisal University, Riyadh 11533, Saudi Arabia; 9 Department of Public Health/Nutrition, The University of Haripur, Haripur 22620, Pakistan; 10 Administration of Pharmaceutical Care, Ministry of Health, Najran 66255, Saudi Arabia; 11 General Surgery Department, Alomran General Hospital, Ministry of Health, Al-Ahsa 36358, Saudi Arabia; 12 Department of Pharmacy, Hereditary Blood Diseases Centre, Ministry of Health, Al-Ahsa 36422, Saudi Arabia; 13 Division of Infectious Diseases and Infection Control, Pediatric Department, Maternity and Children Hospital, Ministry of Health, Al-Ahsa 36422, Saudi Arabia; 14 Division of Infection Control, Maternity and Children Hospital, Ministry of Health, Al-Ahsa 36422, Saudi Arabia; 15 Pharmacy Department, Prince Saud Bin Jalawi Hospital, Ministry of Health, Al-Ahsa 36424, Saudi Arabia; 16 Pharmacy Department, Aljafr General Hospital, Ministry of Health, Al-Ahsa 7110, Saudi Arabia; 17 Primary Care Medicine, Al-Ahsa Health Cluster, Ministry of Health, Al-Ahsa 36421, Saudi Arabia; 18 Nutrition Department, Maternity and Children Hospital, Ministry of Health, Najran 66211, Saudi Arabia; 19 Pharmacy Department, New Najran General Hospital, Ministry of Health, Najran 66244, Saudi Arabia; 20 Department of Chemistry, Government College of Engineering, Keonjhar 758002, Odisha, India; 21 Research Center, Almoosa Specialist Hospital, Al-Ahsa 36342, Saudi Arabia; 22 College of Nursing, Princess Norah Bint Abdulrahman University, Riyadh 11564, Saudi Arabia; 23 School of Nursing, Wollongong University, Wollongong, NSW 2522, Australia; 24 Department of Nursing, Prince Sultan Military College, Dharan 34313, Saudi Arabia

**Keywords:** children, COVID-19, intestinal, intussusception, invagination, meta-analysis, obstruction, pediatric, SARS-CoV-2, systematic review

## Abstract

Background: Intussusception (ISN) post-COVID-19 infection in children is rare but can occur. SARS-CoV-2 may play a role in the pathogenesis of ISN and trigger immune activation and mesenteric adenitis, which predispose peristaltic activity to “telescope” a proximal bowel segment into the distal bowel lumen. Objectives: To estimate the prevalence of SARS-CoV-2 infection in ISN children and analyze the demographic parameters, clinical characteristics and treatment outcomes in ISN pediatric patients with COVID-19 illness. Methods: We performed this systematic review following the Preferred Reporting Items for Systematic Reviews and Meta-Analyses (PRISMA). Studies reporting on the incidence of ISN post-SARS-CoV-2 infection in children, published from 1 December 2019 until 1 October 2022, in PROQUEST, MEDLINE, EMBASE, PUBMED, CINAHL, WILEY ONLINE LIBRARY, SCOPUS and NATURE, with a restriction to articles available in the English language, were included. Results: Of the 169 papers that were identified, 34 articles were included in the systematic review and meta-analysis (28 case report, 5 cohort and 1 case-series studies). Studies involving 64 ISN patients with confirmed COVID-19 (all patients were children) were analyzed. The overall pooled proportions of the ISN patients who had PCR-confirmed SARS-CoV-2 infection was 0.06% (95% CI 0.03 to 0.09, n = 1790, four studies, *I*^2^ 0%, *p* = 0.64), while 0.07% (95% CI 0.03 to 0.12, n = 1552, three studies, *I*^2^ 0%, *p* = 0.47) had success to ISN pneumatic, hydrostatic and surgical reduction treatment and 0.04% (95% CI 0.00 to 0.09, n = 923, two studies, *I*^2^ 0%, *p* = 0.97) had failure to ISN pneumatic, hydrostatic and surgical reduction treatment. The median patient age ranged from 1 to 132 months across studies, and most of the patients were in the 1–12 month age group (n = 32, 50%), *p* = 0.001. The majority of the patients were male (n = 41, 64.1%, *p* = 0.000) and belonged to White (Caucasian) (n = 25, 39.1%), Hispanic (n = 13, 20.3%) and Asian (n = 5, 7.8%) ethnicity, *p* = 0.000. The reported ISN classifications by location were mostly ileocolic (n = 35, 54.7%), and few children experienced ileo-ileal ISN (n = 4, 6.2%), *p* = 0.001. The most common symptoms from ISN were vomiting (n = 36, 56.2%), abdominal pain (n = 29, 45.3%), red currant jelly stools (n = 25, 39.1%) and blood in stool (n = 15, 23.4%). Half of the patients never had any medical comorbidities (n = 32, 50%), *p* = 0.036. The approaches and treatments commonly used to manage ISN included surgical reduction of the ISN (n = 17, 26.6%), pneumatic reduction of the ISN (n = 13, 20.2%), antibiotics (n = 12, 18.7%), hydrostatic reduction of the ISN (n = 11, 17.2%), laparotomy (n = 10, 15.6%), intravenous fluids (n = 8, 12.5%) and surgical resection (n = 5, 7.8%), *p* = 0.051. ISN was recurrent in two cases only (n = 2, 3.1%). The patients experienced failure to pneumatic (n = 7, 10.9%), hydrostatic (n = 6, 9.4%) and surgical (n = 1, 1.5%) ISN treatment, *p* = 0.002. The odds ratios of death were significantly higher in patients with a female gender (OR 1.13, 95% CI 0.31–0.79, *p* = 0.045), Asian ethnicity (OR 0.38, 95% CI 0.28–0.48, *p* < 0.001), failure to pneumatic or surgical ISN reduction treatment (OR 0.11, 95% CI 0.05–0.21, *p* = 0.036), admission to ICU (OR 0.71, 95% CI 0.83–1.18, *p* = 0.03), intubation and placement of mechanical ventilation (OR 0.68, 95% CI 0.51–1.41, *p* = 0.01) or suffering from ARDS (OR 0.88, 95% CI 0.93–1.88, *p* = 0.01) compared to those who survived. Conclusion: Children with SARS-CoV-2 infection are at low risk to develop ISN. A female gender, Asian ethnicity, failure to ISN reduction treatment (pneumatic or surgical), admission to ICU, mechanical ventilation and suffering from ARDS were significantly associated with death following ISN in pediatric COVID-19 patients.

## 1. Introduction

**Figure 1:**
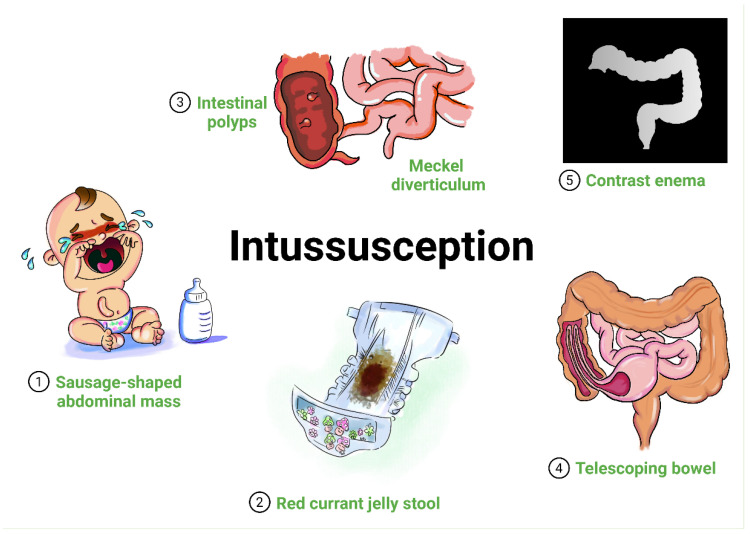
Graphical representation of ISN in children. ISN classically presents in an infant or toddler with (1) sudden onset of intermittent, severe, and progressive abdominal pain and palpable sausage-shaped abdominal mass, and/or (2) red currant jelly stool. ISN may possibly be due to (3) lead points (such as intestinal polyps or Meckel diverticulum). ISN refers to (4) the invagination (telescoping) of a part of the intestine into a more distal segment (proximal segment is known as the intussusceptum and the distal segment into which it telescopes is known as the intussuscipiens). Radiography findings may reveal a (5) lack of perfusion in the intussusceptum, indicating the development of ischemia.

Severe acute respiratory syndrome coronavirus 2 (SARS-CoV-2) infection in children may be underreported, as most cases of coronavirus disease 2019 (COVID-19) in the pediatric population are mild or asymptomatic, but a small number of individuals may develop severe disease, requiring intensive care admission and/or mechanical ventilation [1]. In addition to the respiratory system, SARS-CoV-2 also infects the gastrointestinal system [2]. Some digestive tract symptoms, such diarrhea, vomiting, loss of appetite, stomach upset or abdominal pain, occur with or before respiratory symptoms in patients with COVID-19, with the highest incidence in pediatric age [3]. Very few sporadic cases of intussusception (ISN) in SARS-CoV-2-infected children have been reported worldwide [4,5,6,7]. ISN, defined as the invagination (telescoping) of a part of the intestine into itself, is a rare condition and considered to be the most common abdominal emergency in early childhood, particularly in children younger than two years of age [8,9]. ISN typically presents between 6 and 36 months of age, and it is the most common cause of intestinal obstruction in this age group [8]. In most episodes, ISN occurs in otherwise healthy and well-nourished children [10]. An increasing body of evidence suggests that viral triggers may play a role in the pathogenesis of ISN, and many common viral infections are associated with ISN, including adenovirus, rotavirus and human herpes simplex virus [11,12]. Children are thought to be susceptible to high peristaltic activity to “telescope” a proximal bowel segment into the distal bowel lumen due to the occurrence of local immune activation and mesenteric adenitis [13]. Refractory abdominal pain or mass, vomiting, bloody stool or red currant jelly stools, and lethargy are common symptoms of ISN [13]. Ultrasound or computerized tomography of the abdomen are used for diagnosis (see Figure 1).

With the nonavailability of comprehensive and updated systematic reviews focusing on the co-occurrence of those two medical conditions, we aimed to estimate the prevalence of ISN in pediatric COVID-19 children and analyze the demographic parameters, clinical characteristics and treatment outcomes in ISN patients with pediatric COVID-19 illness, with larger and better-quality data. Because ISN is a very rare phenomenon in adults and occurs mostly in children, the relative odds of ISN coexisting in adult COVID-19 patients was not included in the meta-analysis. We expect our review to provide clinicians with a thorough understanding of the infrequent concurrent occurrence of those two medical conditions in children.

### Aim of the Study

This systematic review and meta-analysis aimed to estimate the prevalence of ISN in COVID-19 children and analyze the demographic parameters, clinical characteristics and treatment outcomes in ISN children with COVID-19 illness.

## 2. Methods

### 2.1. Design

We performed this systematic review following the recommendations of the Preferred Reporting Items for Systematic Reviews and Meta-Analyses guidelines (PRISMA) [14]. We searched for observational studies published from 1 December 2019 until 1 October 2022, in PROQUEST, MEDLINE, EMBASE, PUBMED, CINAHL, WILEY ONLINE LIBRARY, SCOPUS and NATURE, with a restriction to articles published in the English language. The following different keywords were combined: (“*COVID-19*” OR “*SARS-CoV-2*” OR “*Severe acute Respiratory Syndrome Coronavirus 2*” OR “*Coronavirus Disease 2019*” OR “*2019 novel coronavirus*”) AND (“*children*” OR “*child*” OR “*paediatric*” OR “*pediatric*” OR “*infant*” OR “*toddler*” OR “*adolescent*” OR “*newborn*”) AND (“*intussusception*” OR “*intestinal obstruction*” OR “*intestinal invagination*”). Articles discussing and reporting the occurrence of ISN in children infected with COVID-19 were selected based on the title and abstract.

### 2.2. Inclusion–Exclusion Criteria

The eligible studies were included based on the following inclusion criteria: (1) published case reports, case-series and cohort studies that focused on COVID-19 in ISN patients that included children as a population of interest; (2) studies of an experimental or observational design reporting the incidence of SARS-CoV-2 infection in pediatric patients with ISN. The exclusion criteria included: (1) editorials, commentaries, reviews and meta-analyses; (2) studies that reported ISN in children with negative SARS-CoV-2 polymerase chain reaction (PCR) tests; (3) studies that reported ISN in adult COVID-19 patients.

### 2.3. Data Extraction

The screening of the papers was performed independently by six reviewers (Saad Alhumaid, Zainab Al Alawi, Abdulrahman A. Alnaim, Mohammed A. Al Ghamdi, Muneera Alabdulqader and Khalid Al Noaim) by screening the titles with abstracts using the selection criteria. Disagreements in the study selection after the full-text screening were discussed; if agreement could not be reached, a third reviewer was involved. We categorized articles as case report, case-series, clinical trials or cohort studies. The following data were extracted from the selected studies: authors; publication year; study location; study design and setting; age; proportion of male patients; patient ethnicity; medical comorbidities; total number of patients and number of ISN patients with positive SARS-CoV-2; ISN classification by location; symptoms from ISN; abnormal laboratory indicators; radiological imaging findings; if patient was admitted to the intensive care unit (ICU), placed on mechanical ventilation and/or suffered acute respiratory distress syndrome (ARDS); treatment given after ISN; if failure of pneumatic, hydrostatic or surgical reduction in patients with ISN occurred; if ISN was recurrent; assessment of study risk of bias; and final treatment outcome (survived or died); and they are noted in Table 1.

### 2.4. Quality Assessment

Two tools were used appropriately to assess the quality of the studies included in this review: (1) Newcastle-Ottawa Scale (NOS) to evaluate the cohort studies (scoring criteria: >7 scores = high-quality, 5–7 scores = moderate quality and <5 scores = low quality) [45]; (2) modified NOS to evaluate the case report and case-series studies (scoring criteria: 5 criteria fulfilled = good, 4 criteria fulfilled = moderate and 3 criteria fulfilled = low) [46]. A quality assessment was conducted by six co-authors (Koblan M. Al mutared, Yameen Ali Almatawah, Ahmed Tawffeq AlOmran and Sarah Mahmoud Al HajjiMohammed), who separately evaluated the possibility of bias using these two tools.

### 2.5. Data Analysis

We examined primarily the proportion of confirmed SARS-CoV-2 infection in patients with ISN. This proportion was further classified based on the success or failure of pneumatic, hydrostatic or surgical treatment to reduce ISN. A hydrostatic or pneumatic reduction failure to ISN was defined as ISN that could not be reduced using, most commonly, a fluoroscopic guide with hydrostatic (saline or contrast) or pneumatic (air) enema [47]. A surgical reduction failure to ISN was defined as ISN that could not be reduced using an operative intervention [47]. A recurrent ISN was defined as the recurrence of ISN after pneumatic, hydrostatic or surgical reduction (occurrence of abdominal pain and the radiologic appearance of an intussuscepted segment) [48]. Nonrecurrent ISN was defined as the cases that were successfully reduced after pneumatic or hydrostatic intervention or surgery without recurrence [48]. Because all of these data were continuous and dichotomous, these data are presented as numbers (percentages) and odds ratios (ORs) for estimating the point estimate, along with 95% confidence intervals (CIs). For prevalence of SARS-CoV-2 infection in ISN children, pooled effect size was illustrated using a forest plot; and to produce wider CIs than a fixed effect model, we used a random effects with the DerSimoniane–Laird model [49]. The Cochran’s test for chi-squared (*χ*^2^) expressed as the Higgins (*I*^2^) were used to measure the statistical heterogeneity [50]. The degrees of heterogeneity were categorized based on the calculated *I*^2^ values: (not significant: 0–<40%; moderate: 30–60%; substantial: 50–90%; and significant: 75–100%) [51]. Univariate and multivariable logistic regression analyses were used to estimate the odds ratios (ORs) and 95% CIs of the association of each demographic parameter and clinical variable with the treatment outcomes (i.e., survived or died) of ISN patients with SARS-CoV-2 infection. All *p*-values were based on two-sided tests, and significance was set at a *p*-value less than 0.05. We used R version 4.1.0 with the packages *finalfit* and *forestplot* for all statistical analyses.

## 3. Results

### 3.1. Study Characteristics and Quality

**Figure 2:**
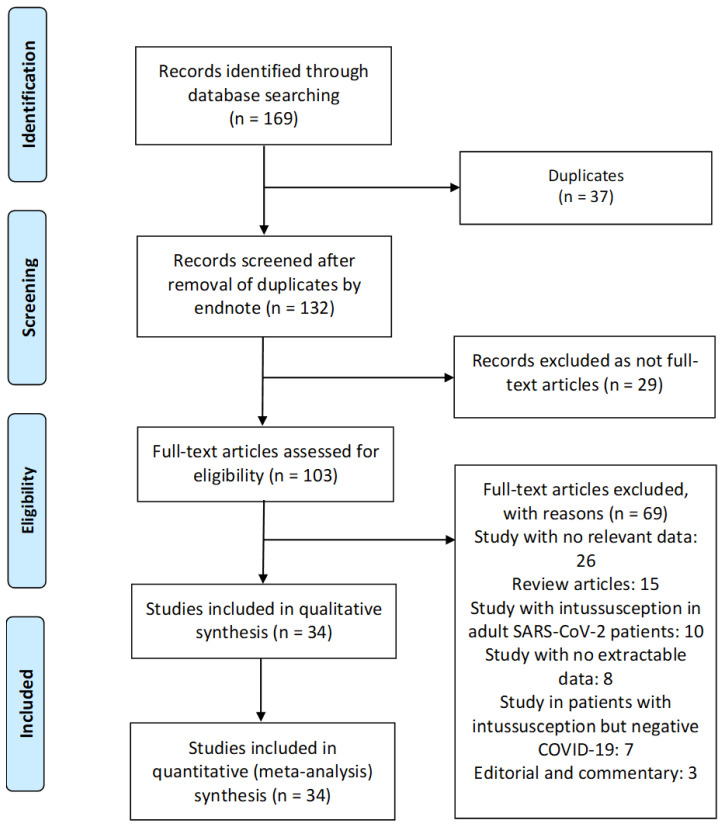
Flow diagram of the literature search and data extraction from studies included in the systematic review and meta-analysis.

We identified 169 manuscripts, and 37 of these articles were duplicates (Figure 2). After removing the duplicates, there were 132 articles left, which we examined by the title and abstract using Endnote, and another 29 irrelevant articles were excluded. Subsequently, 103 articles remained, which were analyzed in full text. In total, 34 studies met our inclusion criteria and reported SARS-CoV-2 infection in pediatric patients with ISN and were included for systematic review and meta-analysis [4,5,6,7,15,16,17,18,19,20,21,22,23,24,25,26,27,28,29,30,31,32,33,34,35,36,37,38,39,40,41,42,43,44]. The detailed characteristics of the included studies are shown in Table 1. There were 28 case report [4,6,7,15,16,17,18,19,21,22,23,24,25,26,27,28,29,30,31,32,33,35,36,37,38,39,40,41], 5 cohort [20,34,42,43,44] and 1 case-series [5] studies. These studies were conducted in United States (n = 9), Italy (n = 4), United Kingdom (n = 3), China (n = 3), India (n = 3), Iran (n = 2), Colombia (n = 2), Jordan (n = 1), Peru (n = 1), Hungary (n = 1), Guatemala (n = 1), Spain (n = 1), Mexico (n = 1), Pakistan (n = 1), and Turkey (n = 1). The majority of the studies were single center [4,5,7,15,16,17,18,19,20,21,22,23,24,25,26,27,29,30,31,32,33,34,35,37,38,39,40,41,43,44], and only four studies were multicenter [6,28,36,42]. Among the 34 included studies, 5 cohort studies were assessed using the NOS: 4 studies were found to be moderate-quality studies (i.e., NOS scores between 5 and 7) and 1 study demonstrated a relatively high quality (i.e., NOS scores > 7). All case reports and case-series studies were assessed for bias using the modified NOS. Twenty-seven studies were deemed to have high methodological quality and two moderate methodological quality (Table 1).

### 3.2. Meta-Analysis of ISN in Pediatric Patients following COVID-19 Infection

**Figure 3:**
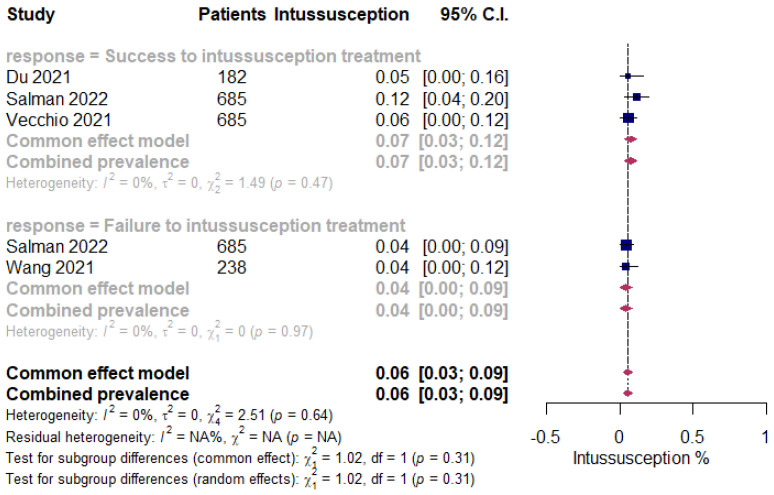
Pooled estimate for the prevalence of ISN in pediatric COVID-19 patients stratified by the failure of pneumatic, hydrostatic or surgical reduction to the ISN.

The overall pooled proportions of pediatric ISN patients who had laboratory-confirmed SARS-CoV-2 infections was 0.06% (95% CI 0.03 to 0.09, n = 1790, 4 studies, *I*^2^ 0%, *p* = 0.64) [20,34,42,43]. The subgroup analysis showed some difference in the rates among all patients (patients who had failure or success to the pneumatic, hydrostatic or surgical reduction of the ISN), and the success to ISN treatment group showed a prevalence of 0.07% (95% CI 0.03 to 0.12, n = 1552, 3 studies, *I*^2^ 0%, *p* = 0.47) [20,34,42], while the failure to ISN treatment group shown a prevalence of 0.04% (95% CI 0.00 to 0.09, n = 923, 2 studies, *I*^2^ 0%, *p* = 0.97) [34,43], respectively (Figure 3).

### 3.3. Demographic and Clinical Characteristics of ISN Pediatric Patients with SARS-CoV-2 Infection

The included studies had a total of 64 ISN patients with confirmed SARS-CoV-2 infection, as detailed in Table 1. Amongst these 64 patients, all patients were children. The median patient age ranged from 1 to 132 months across the studies. Most of the patients were in the 1-12 months age group (n = 32, 50%), *p* = 0.001 [4,5,6,7,15,16,17,18,20,22,23,25,26,27,28,29,30,31,35,36,38,39,40,41,43]. There was an increased male predominance in ISN patients diagnosed with SARS-CoV-2 in most of the studies (n = 41, 64.1%, *p* = 0.000) [4,7,15,16,17,19,21,22,23,25,26,27,28,29,30,31,33,34,36,37,38,39,40,42], and the majority of the patients belonged to White (Caucasian) (n = 25, 39.1%), Hispanic (n = 13, 20.3%) and Asian (n = 5, 7.8%) ethnicity, *p* = 0.000 [4,5,6,18,19,20,21,22,23,24,25,26,27,30,31,32,34,35,36,38,39,40,41,42,43,44]. The reported ISN classification by location were mostly ileocolic (n = 35, 54.7%) [4,6,7,15,17,18,19,20,21,22,23,24,25,26,27,28,29,30,31,32,34,36,37,38,40], and few patients experienced ileo-ileal ISN (n = 4, 6.2%) [16,39,42,44], *p* = 0.001. Most patients were diagnosed for ISN through symptoms, radiological imaging and nonoperative reduction using the hydrostatic (contrast or saline) or pneumatic (air) enema, as both procedures are diagnostic and therapeutic [4,5,6,7,15,16,17,18,19,20,21,22,23,24,25,26,27,28,29,30,31,32,33,34,35,36,37,38,39,40,41,42,43,44]. The most common symptoms from ISN were vomiting (n = 36, 56.2%) [4,5,6,7,16,17,18,19,20,21,22,23,25,26,27,28,30,33,34,35,36,37,38,42,43], abdominal pain (n = 29, 45.3%) [4,15,16,18,19,21,22,24,26,29,30,31,32,33,34,37,38,39,42,44], red currant jelly stools (n = 25, 39.1%) [5,6,7,16,17,22,23,25,27,28,29,30,31,34,35,38,40,42], blood in stool (n = 15, 23.4%) [4,5,15,17,25,26,34,36,40,41,43,44], anorexia (n = 9, 14.1%) [4,18,19,23,29,30,36,37,40], irritability (n = 8, 12.5%) [18,22,26,28,29,38], abdominal tenderness (n = 8, 12.5%) [16,17,21,24,25,33,44], crying (n = 8, 12.5%) [5,6,16,18,24,28,29,39], abdominal distension (n = 7, 10.9%) [5,6,15,17,25,28,33], dehydration (n = 7, 10.9%) [4,7,17,21,30,36,37], lethargy (n = 7, 10.9%) [6,7,24,30,36,40], pallor (n = 6, 9.4%) [16,22,28,29,30,37] and diarrhea (n = 6, 9.4%) [25,30,33,41,43,44], *p* = 0.179. Imaging detected acute ISN in most of the patients (n = 35, 57.8%) [4,5,6,7,15,16,17,18,19,20,21,22,23,24,25,26,27,28,29,30,31,32,33,34,35,36,37,38,39,40,41,43,44]; enlarged lymph nodes (n = 6, 9.4%) [18,21,24,25,26,36], the classic manifestation of ISN “target sign” (n = 4, 6.2%) [17,27,39,41], intestinal necrosis (n = 4, 6.2%) [5,33,36,43] and doughnut-shaped mass (n = 2, 3.1%) [32,40] were reported, *p* = 0.237. The most common laboratory findings were high C-reactive protein (n = 14, 21.9%) [5,6,16,18,19,20,21,22,27,28,32,41,43,44], high D-dimer (n = 9, 14.1%) [5,15,16,21,22,28,29,30,43], low hemoglobin (n = 6, 9.4%) [7,16,22,27,28,29], raised procalcitonin (n = 5, 7.8%) [5,15,40,43,44], high leukocytes (n = 4, 6.2%) [19,37,40,41], decreased lymphocytes (n = 4, 6.2%) [4,5,33,44], high lactate dehydrogenase (n = 4, 6.2%) [16,20,24,43], and high ferritin (n = 4, 6.2%) [5,16,22,44], *p* = 0.063. Half of the patients never had any medical comorbidities (n = 32, 50%) [4,5,6,7,15,16,17,18,19,20,22,23,24,25,26,27,28,29,30,31,32,33,35,38,39,40,41,43,44]; however, a medical history was not reported for a high number of patients (n = 15, 23.4%) [34,42], *p* = 0.036. The ten most used approaches and treatments to manage ISN were surgical reduction of the ISN (n = 17, 26.6%) [6,15,16,18,19,26,28,34,35,42,43], pneumatic reduction of the ISN (n = 13, 20.2%) [6,7,17,25,29,32,34,35,36,38], antibiotics (n = 12, 18.7%) [5,6,16,17,18,20,21,22,29,33,37,43], hydrostatic reduction of the ISN (n = 11, 17.2%) [4,18,23,24,26,27,30,37,39,40], laparotomy (n = 10, 15.6%) [18,19,21,26,30,31,36,40], intravenous fluids (n = 8, 12.5%) [7,16,17,18,21,24,37,40], oxygen supplementation (n = 6, 9.4%) [5,16,20,21,22,24], analgesics (n = 5, 7.8%) [19,21,22,24,29], surgical resection (n = 5, 7.8%) [5,21,22,30,31], and steroids (n = 5, 7.8%) [5,16,37,43,44], *p* = 0.051. ISN was reported to be recurrent in two cases only (n = 2, 3.1%) [38,39], *p* = 0.140. Patients experienced failure to pneumatic (n = 7, 10.9%) [6,34,35,36,38], hydrostatic (n = 6, 9.4%) [18,26,30,39,40] and surgical (n = 1, 1.5%) [43] ISN treatment, *p* = 0.002. There were patients who were admitted to the intensive care units (n = 9, 14.1%, *p* = 0.000) [5,6,16,20,21,30,31,36,43], intubated and placed on mechanical ventilation (n = 6, 9.4%, *p* = 0.000) [5,6,20,21,36,43] and suffered acute respiratory distress syndrome (n = 6, 9.4%, *p* = 0.000) [5,6,16,20,36,43].

### 3.4. Treatment Outcome and Predictors of Mortality in Pediatric COVID-19 Patients with ISN

The patients were stratified based on the treatment outcome (i.e., mortality or survival). A summary of the demographic, ISN classification by location, imaging findings, symptoms from ISN, laboratory parameters, comorbidities, treatment received and medical complications with regards to final treatment outcome in 64 patients who had either survived (n = 43) [4,6,7,15,16,17,18,19,21,22,23,24,25,26,27,28,29,30,31,32,33,34,35,36,37,38,39,40,41,42,44] or died (n = 4) [5,6,20,43] is shown in Table 2.

Those patients who died were all in the 1 month to <1 year age group (1 month to less than 1 year: 100% vs. 65.1%, *p* = 0.001) [5,6,20,43], females (female gender: 100% vs. 32.5%, *p* = 0.000) [5,6,20,43], and had an Asian ethnicity (Asian ethnicity: 100% vs. 2.3%, *p* = 0.000) [5,6,20,43]. Patients were likely to die from the most common type of ISN by location (ileocolic ISN: 53.1% vs. 50%, *p* = 0.001) [6,20]. ISN patients who shown radiological images of acute ISN (100% vs. 76.7%, *p* = 0.237) and intestinal necrosis (50% vs. 4.6%, *p* = 0.237) had a higher mortality compared to those ISN patients who did not show those imaging findings [5,6,20,43]. The most common ISN symptoms in patients with SARS-CoV-2 infection in whom mortality was reported were the red currant jelly stools (n = 2, 50%) [5,6], blood in stool (n = 2, 50%) [5,43], crying (n = 2, 50%) [5,6], abdominal distension (n = 2, 50%) [5,6], diarrhea (n = 1, 25%) [43] and lethargy (n = 1, 25%) [6], *p* = 0.179. Patients who died were more likely to present with higher levels of the following: C reactive protein (100% vs. 23.2%) [5,6,20,43], D-dimer (50% vs. 16.3%) [5,43], raised procalcitonin (50% vs. 7%) [5,43], lactate dehydrogenase (50% vs. 4.6%) [20,43], high interleukin-6 (50% vs. 2.3%) [5,43], and blood urea nitrogen (50% vs. 0) [20,43]. Unexpectedly, all patients who died had no medical history (100% vs. 65.1%, *p* = 0.036) [5,6,20,43]. ISN patients infected with SARS-CoV-2 who received antibiotics (100% vs. 18.6%) [5,6,20,43], surgical reduction (50% vs. 34.9) [6,43], oxygen supplementation (50% vs. 9.3%) [5,20], steroids (50% vs. 7%) [5,43], continuous renal replacement therapy (50% vs. 0%) [5,43], plasma exchange (50% vs. 0%) [5,43], interferon (50% vs. 0%) [5,20], antivirals (50% vs. 0%) [5,43], or intravenous immunoglobulin (50% vs. 2.3) [5,43] had higher mortality compared to ISN patients with SARS-CoV-2 who never had those treatments, *p* = 0.051. All patients who died had no recurrent ISN (100% vs. 95.3%, *p* = 0.140) [5,6,20,43]; however, patients who had no failure to surgical, pneumatic and hydrostatic ISN reduction treatment (50% vs. 53.5%) were more likely to die, *p* = 0.002 [5,20]. The mortality rate was significantly very high in ISN patients infected with SARS-CoV-2 who were admitted to the ICU (100% vs. 11.6%, *p* = 0.000) [5,6,20,43], intubated and placed on mechanical ventilation (100% vs. 4.6%, *p* = 0.000) [5,6,20,43] and/or suffered ARDS (100% vs. 4.6%, *p* = 0.000) [5,6,20,43]. Other investigations of the cases included in this review are indicated in Table 2.

The potential determining variables associated in the survival and death groups were analyzed through binary logistic regression analysis and are shown in Table 3. The patients in the 1 month to <1 year age group (OR 0.42, 95% CI 0.1–0.33, *p* = 0.04); female gender (OR 0.74, 95% CI 0.36–0.52, *p* < 0.001); ISN patients infected with COVID-19 who came from Asia (OR 0.36, 95% CI 0.26–0.45, *p* < 0.001); patients who experienced disseminated intravascular coagulopathy (OR 1, 95% CI 0.35–1.65, *p* = 0.003) or multiple organ failure (OR 1, 95% CI 0.35–1.65, *p* = 0.003); failed pneumatic or surgical treatment to ISN reduction (OR 0.16, 95% CI 0.06–0.27, *p* = 0.002); admitted to ICU (OR 0.53, 95% CI 0.45-0.6, *p* < 0.001), intubated and mechanically ventilated (OR 0.84, 95% CI 0.79–0.9, *p* < 0.001); suffered ARDS (OR 0.75, 95% CI 0.68-0.81, *p* < 0.001); or received antibiotics (OR 0.31, 95% CI 0.02–0.59, *p* = 0.03), antivirals (OR 1, 95% CI 0.61–1.39, *p* < 0.001), continuous renal replacement therapy (OR 1, 95% CI 0.55-1.45, *p* < 0.001), dopamine or inotropes (OR 0.5, 95% CI 0.05–0.95, *p* = 0.03), interferon (OR 1, 95% CI 0.55–1.45, *p* < 0.001), intravenous immunoglobulin (OR 0.75, 95% CI 0.39-1.11, *p* < 0.001), Ladd’s procedure (OR 0.5, 95% CI 0.05–0.95, *p* = 0.03), oxygen supplementation (OR 0.33, 95% CI 0.61–1.39, *p* = 0.04), plasma exchange (OR 1, 95% CI 0.55–1.45, *p* < 0.001), and steroids (OR 0.5, 95% CI 0.17–0.83, *p* < 0.001) were associated with an increased odds ratio for death (Table 3). These variables were considered needing further evaluation and, thus, were included in multivariate regression analysis. Nevertheless, multivariate analysis confirmed female gender (OR 1.13, 95% CI 0.31–0.79, *p* = 0.045); ISN patients with SARS-CoV-2 infection located in Asia (OR 0.38, 95% CI 0.28–0.48, *p* < 0.001); patients who failed pneumatic or surgical reduction to the ISN treatment (OR 0.11, 95% CI 0.05–0.21, *p* = 0.036); patients admitted to ICU (OR 0.71, 95% CI 0.83–1.18, *p* = 0.03), intubated and placed on mechanical ventilation (OR 0.68, 95% CI 0.51–1.41, *p* = 0.01); or suffered from ARDS (OR 0.88, 95% CI 0.93–1.88, *p* = 0.01) were significantly associated with increased death (Table 3).

## 4. Discussion

In this small systematic review and meta-analysis, we included 64 pediatric patients with PCR-confirmed SARS-CoV-2 infection from 34 observational studies to estimate the incidence of ISN in children with COVID-19. Linking between COVID-19 and ISN and establishing the relationship between them may help avoid diagnostic delays and allow for the development of more specific and efficient ways of ISN prevention and therapy. As expected, the overall incidence of ISN in pediatric patients infected with SARS-CoV-2 was very low (0.06%). The Incidence of ISN in pediatric COVID-19 patients who had a failure to pneumatic, hydrostatic or surgical reduction treatment compared to ISN patients with COVID-19 in whom intestinal obstruction was reduced successfully with pneumatic, hydrostatic or surgical interventions was even almost twofold lower in this group of ISN patients (0.04% vs. 0.07%). ISN is a rare form of intestinal obstruction in which a segment of the bowel prolapses into a more distal portion [52]. It can be argued that the prevalence of ISN in the pediatric population decreased during the COVID-19 pandemic, an issue that can be linked to COVID-19 containment policies and public information campaigns [53,54], which resulted in the improvement of complying with infection control and prevention measures by children (higher adherence to mask wearing and hand washing) [55,56,57,58] and reduced the transmission of bacterial and viral pathogens in many countries worldwide [59]. Our systematic review showed different results from previous case reports in which only a limited preliminary assessment of the potential size and scope of the available ISN cases among SARS-CoV-2-infected children was performed [4,17,19,25,28,30,41]. We were able to report the first pooled effect size of ISN prevalence in hospitalized pediatric COVID-19 patients because this review is more comprehensive and included a total of 34 studies [4,5,6,7,15,16,17,18,19,20,21,22,23,24,25,26,27,28,29,30,31,32,33,34,35,36,37,38,39,40,41,42,43,44], including a total of 64 COVID-19 children. The inclusion of 22 recently published studies [15,16,18,20,21,22,23,24,26,31,32,33,34,35,36,37,38,39,40,42,43,44] contributed to the refinement of the estimate of the pooled prevalence of ISN contributing to intestinal obstruction in COVID-19 pediatric patients. We estimated a comparable incidence of ISN among COVID-19 children (incidence: 40 to 70 cases per 100,000) to the previous studies that evaluated the yearly mean prevalence of ISN in children from Switzerland (incidence: 38 cases per 100,000) [60], Australia (incidence: 71 cases per 100,000) [61], the United Kingdom (incidence: 66 cases per 100,000) [62] and Singapore (incidence: 60 cases per 100,000) [63].

Analyzing the demographic and clinical characteristics of the ISN cases with COVID-19, we found that the age of presentation, preponderance in males, lack of previous medical history, location of affected intestinal segments, predominant ethnicity and symptoms from intestinal obstruction were maintained, like most reported cases of ISN. Our results align with some prior research that identified that ISN cases were more incident in the 1 to 12 month age group and in males [64,65], and most ISN episodes occurred in otherwise healthy and well-nourished children [66,67] and commonly involved the ileocecal junction (i.e., ileocolic type) [68,69]. We found that the development of COVID-19 in ISN children was highest in patients of White (Caucasian) and Hispanic ethnicity, and compared to whites, Hispanics had a two-fold lowered risk of ISN (39% and 20%, respectively). These findings are consistent with previous observations that estimated rates of ISN among Caucasian children are higher than among Hispanics and Asians [70,71]. Whether differences in factors such as genetics, diet or environment could explain this increased risk remains unclear. However, the differences in the frates of ISN by ethnicity could possibly be explained by low socioeconomic status [72], difference in access to health care or lack of accessibility to medical treatment [73], health care-seeking behavior [74] or lack of awareness among parents when their child presents with symptoms of ISN leading them to seek medical attention [75]. ISN classically presents in an infant or toddler with sudden onset of intermittent, severe and progressive abdominal pain, accompanied by inconsolable crying often with vomiting, palpable sausage-shaped abdominal mass, red currant jelly stools, anorexia, dehydration, irritability, abdominal tenderness, lethargy and pallor [76,77]. It is important to know that ISN can be an unusual manifestation of COVID-19. It is even more imperative to suspect ISN when a COVID-19 child presents with abdominal pain, vomiting, firm and palpable mass or blood in the stool [76,77]. In these cases, timely diagnosis is crucial for adequate treatment and a good prognosis [30]. A delay in diagnosis secondary to delayed presentation will lead to a delay in providing the adequate noninvasive treatment through enema reduction and an increased risk of treatment failure [78]. The COVID-19 pandemic has been reported to lead to the diagnostic delay of ISN and the deterioration of patients’ clinical manifestations [79]; a high rate of bowel resection and morbidity was a consequence of delayed ISN presentation [80] and might have resulted in a lower number of ISN patients visiting the emergency department [81] or in some serious illnesses such as ISN remaining undiagnosed [82].

ISN may be the result of anatomical causes, associated diseases and, pertinent to cases included in this review, viral infections [83,84]. However, 75% of ISN cases occurred due to the lack of an identifiable lead point (i.e., idiopathic) [84]. One accepted theory regarding the pathogenesis of ISN and its correlation with viral infection is based on Peyer’s patch swelling and lymph node hypertrophy acting as lead points. Adenovirus, rotavirus, norovirus, human herpes virus 6, astrovirus, enterovirus and cytomegalovirus, along with some parasites, have been identified as agents that can cause ISN [85,86]. SARS-CoV-2 has been known to infect cells via angiotensin-converting enzyme 2 (ACE-2) receptors and the transmembrane protease serine 2 (TMPRSS2) enzyme, which are highly expressed in the human’s digestive system and mediate SARS-CoV-2 entry into the intestinal epithelial cells [87,88]. It is reasonable to hypothesize that any virus capable of triggering an enteric inflammatory response could produce an ISN in a vulnerable host, and inflammation of the small intestine and associated lymphatic hyperplasia from SARS-CoV-2 infection may result in ISN [36].

Depending on the duration of ISN illness and associated vomiting and blood loss, laboratory investigations for most of the ISN cases included in our review reflected dehydration, anemia, leukocytosis, or a combination of these [15,16,18,19,21,24,37,41,43]. However, laboratory abnormalities are not specific for ISN. Several imaging modalities can assist in the diagnosis of ISN [89]. The initial assessment should include plain abdominal radiographs to exclude perforation [90], which, if present, requires operative management rather than nonoperative reduction [91]; however, plain films have low sensitivity for the detection of ISN (<48%) [92]. Therefore, ultrasonography is the method of choice to detect ISN, and the sensitivity and specificity of this technique approaches 100% [93,94]. Ultrasound is better at characterizing pathological lead points than fluoroscopic techniques, can be used to monitor the success of a reduction procedure, and does not expose the patient to radiation [89]. A positive ultrasound shows evidence of a target sign (also called a doughnut sign or bull’s eye sign), which represents layers of the intestine within the intestine and embodies ISN [94]. ISN can be recognized on computerized tomography, which may also identify the cause [95]; however, computerized tomography cannot be used to reduce the ISN [96], can be time consuming in children who may require sedation [97] and also exposes the patients to significant radiation [92].

Most of the ileocolic ISN patients who were hemodynamically stable and had no evidence of intestinal perforation were treated with nonoperative reduction techniques (i.e., pneumatic and/or hydrostatic). Pneumatic or hydrostatic pressure by enema is the treatment of choice to reduce ISN and has high success rates [98,99]. Surgical reduction may also be necessary if nonoperative reduction fails to reduce the ISN [100]. Consistent with recommendations from the American Pediatric Surgical Association [101], we found only a few children who received prophylactic antibiotics prior to or during nonoperative reduction, as there is no evidence that this practice is beneficial, likely because bacteremia and perforation are rare, except for in children with hemodynamic instability or critical illness [101]. Few of the ISN cases that had an ileo-ileal type were managed without nonoperative reduction, because the involvement of small bowel ISN is less likely to respond to pneumatic or hydrostatic reduction and more likely to reduce spontaneously (follow-up and bowel rest) [102].

We found that the mortality rate in ISN children infected with COVID-19 was significantly high in female patients with Asian ethnicity, in line with findings in previous reports that showed that females and children of Asian ethnicity with ISN were significantly associated with death [103,104] and in contradiction with data from a national study that examined trends in ISN-associated deaths among United States infant from 1979 to 2007, demonstrating that death was lower in females [105]. The difference in the mortality due to the fact of ISN based on gender has not been described in the literature previously; however, this might be attributed mainly to the differences in the severity of the ISN illness and/or inclusion criteria, or the level of health care infrastructure and general care-seeking practices in low- and middle-income countries [106,107]. Nevertheless, mortality in association with ISN is quite low in most parts of the world (<1%) [104]. Mortality in COVID-19 cases with ISN included in our review were complicated by multi-inflammatory systemic infection in children (i.e., cytokine storm), and the patients died due to the fact of subsequent multiple organ failure induced by the viral invasion [108].

### Limitations of the Study

First, while all of the evidence discussed was based on one case-series, a few cohorts and many case reports, many of these were small and performed in single centers and are not necessarily generalizable to SARS-CoV-2-infected children with ISN. Second, to assess factors associated with mortality in ISN children infected with SARS-CoV-2, a larger cohort of patients is needed. Last, the study was not registered in Prospero, an international prospective register of systematic reviews, as this might have added extra work, and the merit was mostly limited to the avoidance of duplication.

## 5. Conclusions

Children with SARS-CoV-2 infection are at low risk to develop ISN and may experience SARS-CoV-2-induced ARDS or pneumonia and need ICU admission and mechanical ventilation. Female gender, Asian ethnicity, failure to ISN reduction treatment (pneumatic or surgical), admission to ICU, mechanical ventilation and suffering from ARDS were significantly associated with death following ISN in pediatric COVID-19 patients. These findings may help to design targeted interventions that raise health care providers’ awareness regarding the risk of intestinal obstruction among infants who present to the emergency department.

## Figures and Tables

**Table 1:** Summary of the characteristics of the included studies with evidence on ISN and COVID-19 in pediatric patients (n = 34 studies), 2020–2022.

Author, Year, Study Location	Study Design, Setting	Age (Months) ^a^	Male, n (%)	Ethnicity	Comorbidities, n	Number of Patients (n = 1828)	Number of SARS-CoV-2 Patients with ISN (n = 64, 3.5%)	ISN Classification by Location AND Symptoms from ISN, n	Laboratory Findings	Imaging	Admitted to ICU, n	Mechanical Ventilation, n	ARDS, n	Treatment, n; If Failure to Pneumatic, Hydrostatic or Surgical Reduction Occurred; If ISN Was Recurrent	Assessment of Study Risk of Bias (Tool Used, Finding); Treatment Outcome
Acharyya et al., 2022 [15], India	Retrospective case report, single center	4	1 (100)	1 Indian	1 No medical history	1	1	1 Ileocolic ISN AND 1 Abdominal pain 1 Abdominal distension 1 Blood in stool 1 Large hematoma in the serous layer of the intestine	1 Raised procalcitonin 1 High D-dimer 1 High prothrombin time 1 High NT-proBNP	1 Acute ISN 1 Multiple air fluid levels	0	0	0	1 Surgical reduction of the ISN No failure (n = 1) No recurrence (n = 1)	(Modified NOS, high) 1 survived
Agarwalla and Jalan 2022 [16], India	Retrospective case report, single center	10	1 (100)	1 Indian	1 No medical history	1	1	1 Ileo-ileal ISN AND 1 Abdominal pain 1 Abdominal tenderness 1 Crying 1 Vomiting 1 Red currant jelly stools 1 Drowsiness 1 Low BP 1 Pallor 1 Tachycardia 1 Low oxygen saturation 1 Cold extremities 1 Peripheral pulses 1 Autonomic dysfunction 1 Irregular heartbeat 1 Ectopic beats	1 Low Hb 1 High CRP 1 High ferritin 1 High D-dimer 1 High LDH	1 Acute ISN	1	0	1	1 Surgical reduction of the ISN 1 Dopamine 1 IV fluids 1 Oxygen supplementation 1 Inotropes 1 Antibiotics 1 Steroids 1 Anticoagulant 1 NGT feeding 1 Removal of surgical drain 1 Dressing No failure (n = 1) No recurrence (n = 1)	(Modified NOS, high) 1 Survived
Athamnah et al., 2021 [17], Jordan	Retrospective case report, single center	2.5	1 (100)	1 Arab	1 No medical history	1	1	1 Ileocolic ISN AND 1 Fever 1 Vomiting 1 Dehydration 1 Blood in stool 1 Abdominal distension 1 Abdominal tenderness 1 Bilious discharge from the NGT 1 Red currant jelly stools	1 Not reported	1 Distal small bowel obstruction and decreased gas in the colon 1 Acute ISN (target sign)	0	0	0	1 Pneumatic reduction of the ISN 1 IV fluids 1 Antibiotics No failure (n = 1) No recurrence (n = 1)	(Modified NOS, high) 1 Survived
Bazuaye-Ekwuyasi et al., 2020 [4], United States	Retrospective case report, single center	9	1 (100)	1 Hispanic	1 No medical history	1	1	1 Ileocolic ISN AND 1 Vomiting 1 Abdominal pain 1 Anorexia 1 Blood in stool 1 Dehydration	1 Ketonuria 1 Proteinuria 1 Decreased lymphocytes	1 Acute ISN 1 Colon cutoff sign of ISN	0	0	0	1 Hydrostatic reduction of the ISN No failure (n = 1) No recurrence (n = 1)	(Modified NOS, high) 1 Survived
Cai et al., 2020 [5], China	Retrospective case-series, single center	10	0 (0)	1 Asian	1 No medical history	5	1	1 Location was not reported AND 1 Crying 1 Restlessness 1 Vomiting 1 Red currant jelly stools 1 Apathy 1 Drowsiness 1 Convulsions 1 Septic shock 1 Multiple organ dysfunction 1 Abdominal distension 1 Blood in stool 1 Coffee dreg-like gastric contents	1 Leukopenia 1 Decreased lymphocytes 1 Thrombocytopenia 1 High CRP 1 Raised procalcitonin 1 High D-dimer 1 High prothrombin time 1 High APTT 1 High ferritin 1 High interleukin-6 1 High interleukin-10 1 Hypoalbuminemia 1 Hyponatremia 1 Hypocalcemia 1 Reduced number of CD3+, CD4+, CD8+ T lymphocytes and CD16 + CD56 natural killer cells	1 Acute ISN 1 Large amount of abdominal dropsy 1 Necrosis of the proximal ileus of the small intestine	1	1	1	1 Steroids 1 IVIG 1 Interferon 1 Antivirals 1 Antibiotics 1 Oxygen supplementation 1 CRRT 1 Plasma exchange 1 Surgical resection of necrotic intestine 1 Dopamine 1 Dobutamine No failure (n = 1) No recurrence (n = 1)	(Modified NOS, high) 1 Died
Castellazzi et al., 2021 [18], Italy	Retrospective case report, single center	10	0 (0)	1 White (Caucasian)	1 No medical history	1	1	1 Ileocolic ISN AND 1 Abdominal pain 1 Irritability 1 Anorexia 1 Crying 1 Vomiting 1 Rectal blood 1 Spiking colicky pain	1 High CRP	1 Acute ISN 1 Increased thickness of the intestinal wall and accompanying mesentery 1 Enlarged lymph nodes	0	0	0	1 Hydrostatic reduction of the ISN 1 Antibiotics 1 IV fluids 1 Surgical reduction of the ISN 1 Laparotomy 1 Ileocecopexy Yes failure (hydrostatic, n = 1) No recurrence (n = 1)	(Modified NOS, high) 1 Survived
Díaz-Ruiz et al., 2022 [19], Peru	Retrospective case report, single center	72	1 (100)	1 Hispanic	1 No medical history	1	1	1 Ileocolic ISN AND 1 Abdominal pain 1 Liquid stools with mucus and no blood 1 Anorexia 1 Chills 1 Vomiting 1 Nausea	1 High CRP 1 High leukocytes	1 Acute ISN	0	0	0	1 Surgical reduction of the ISN 1 Laparotomy 1 Appendectomy 1 Analgesics No failure (n = 1) No recurrence (n = 1)	(Modified NOS, high) 1 Survived
Du et al., 2021 [20], China	Retrospective cohort, single center	10	0 (0)	1 Asian	1 No medical history	182	1	1 Ileocolic ISN AND 1 Nausea 1 Vomiting 1 Multiple organ dysfunction 1 Intestinal necrosis 1 Septic shock 1 DIC	1 High CRP 1 High LDH 1 High BUN	1 Acute ISN	1	1	1	1 Interferon 1 Antibiotics 1 Oxygen supplementation No failure (n = 1) No recurrence (n = 1)	(NOS, 7) 1 Died
Fadgyas et al., 2022 [21], Hungary	Retrospective case report, single center	90	1 (100)	1 White (Caucasian)	1 Left-sided renal agenesis 1 Developmental delay 1 Meningism	1	1	1 Ileocolic ISN AND 1 Abdominal tenderness 1 Headache 1 Vomiting 1 Facial petechiae 1 Dehydration 1 Abdominal pain 1 Low BP	1 High CRP 1 High WBCs 1 High neutrophils 1 High D-dimer 1 Raised liver enzymes 1 Raised bilirubin 1 High troponin	1 Enlarged mesenteric lymph nodes 1 Acute ISN 1 Very dilated, fluid- and gas-filled small bowel loops 1 Meckel’s diverticulum 1 Bowel obstruction	1	1	0	1 Antibiotics 1 Analgesics 1 IV fluids 1 Oxygen supplementation 1 Laparotomy 1 Surgical resection 1 Anastomosis No failure (n = 1) No recurrence (n = 1)	(Modified NOS, high) 1 Survived
García et al., 2021 [22], Guatemala	Retrospective case report, single center	6	1 (100)	1 Hispanic	1 No medical history	1	1	1 Ileocolic ISN AND 1 Irritability 1 Vomiting 1 Red currant jelly stools 1 Pallor 1 Abdominal pain	1 Low Hb 1 High D-dimer 1 High CRP 1 High ferritin	1 Acute ISN 1 Dilation of intestinal loops 1 Poor gas distribution	0	0	0	1 Antibiotics 1 Analgesics 1 Surgical resection 1 Oxygen supplementation 1 Total parenteral nutrition No failure (n = 1) No recurrence (n = 1)	(Modified NOS, high) 1 Survived
Guerrón and Figueroa 2021 [23], Colombia	Retrospective case report, single center	5	1 (100)	1 Hispanic	1 No medical history	1	1	1 Ileocolic ISN AND 1 Vomiting 1 Red currant jelly stools 1 Absence of bowel sounds 1 Anorexia	1 Not reported	1 Acute ISN 1 Significant colonic parietal thickening	0	0	0	1 Hydrostatic reduction of the ISN No failure (n = 1) No recurrence (n = 1)	(Modified NOS, high) 1 Survived
Hyun et al., 2022 [24], United States	Retrospective case report, single center	20	0 (0)	1 Hispanic	1 No medical history	1	1	1 Ileocolic ISN AND 1 Weakness 1 Lethargy 1 Abdominal pain 1 Abdominal tenderness 1 Increased fussiness 1 Crying 1 Facial cyanosis 1 Cold extremities 1 Perception of a firm and painful mass 1 Low oxygen saturation	1 High WBCs 1 High neutrophils 1 High monocytes 1 Hypokalemia 1 High LDH	1 Acute ISN 1 Concentric rings of the bowel 1 Enlarged lymph nodes	0	0	0	1 Analgesics 1 Oxygen supplementation 1 IV fluids 1 Hydrostatic reduction of the ISN No failure (n = 1) No recurrence (n = 1)	(Modified NOS, high) 1 Survived
Khan et al., 2021 [25], United States	Retrospective case report, single center	2	1 (100)	1 White (Caucasian)	1 No medical history	1	1	1 Ileocolic ISN AND 1 Abdominal tenderness 1 Vomiting 1 Diarrhea 1 Blood in stool 1 Abdominal distension 1 Red currant jelly stools	1 Not reported	1 Acute ISN 1 Enlarged lymph nodes	0	0	0	1 Pneumatic reduction of the ISN No failure (n = 1) No recurrence (n = 1)	(Modified NOS, high) 1 Survived
Leiva et al., 2022 [26], United States	Retrospective case reports, single center	7 and 9	1 (50)	2 Whites (Caucasians)	1 No medical history	2	2	2 Ileocolic ISN AND 2 Abdominal pain 2 Irritability 1 Vomiting 2 Blood in stool	1 Not reported	2 Acute ISN 1 Hypertrophied lymph nodes	0	0	0	2 Hydrostatic reductions of the ISN 2 Surgical reductions of the ISN 2 Laparotomy Yes failure (hydrostatic, n = 2) No recurrence (n = 2)	(Modified NOS, high) 2 Survived
Makrinioti et al., 2020 [6], United Kingdom	Retrospective case reports, multicenter	10 and 10	0 (0)	1 White (Caucasian) and 1 Asian	2 No medical history	2	2	2 Ileocolic ISN AND 1 Crying 2 Vomiting 2 Red currant jelly stools 1 Abdominal distension 1 Absence of bowel sounds 1 Peritonitis 1 Ascites 1 Swelling of the small intestinal wall 1 Lethargy 1 DIC	2 High CRP	1 Acute ISN	1	1	1	2 Pneumatic reductions of the ISN 1 Laparotomy 1 Defunctioning ileostomy 1 Antibiotics 1 Inotropes 1 Surgical reduction of the ISN 1 Ladd’s procedure No failure (n = 1) Yes failure (pneumatic, n = 1) No recurrence (n = 2)	(Modified NOS, high) 1 Survived 1 Died
Martínez-Castaño et al., 2020 [27], Spain	Retrospective case report, single center	6	1 (100)	1 White (Caucasian)	1 No medical history	1	1	1 Ileocolic ISN AND 1 Vomiting 1 Abdominal cramps 1 Red currant jelly stools 1 Anemia	1 Low Hb 1 High CRP 1 High WBCs	1 Acute ISN (target sign)	0	0	0	1 Hydrostatic reduction of the ISN No failure (n = 1) No recurrence (n = 1)	(Modified NOS, high) 1 Survived
Mercado-Martínez et al., 2021 [28], Mexico	Retrospective case reports, multicenter	8 and 7	1 (50)	2 Hispanics	2 No medical history	2	2	2 Ileocolic ISN AND 1 Crying 2 Vomiting 2 Red currant jelly stools 2 Irritability 1 Pallor 1 Tachycardia 1 Abdominal distension 1 Decreased peristalsis 1 Painful on deep palpation in mesogastrium 1 Perception of a firm and painful mass 2 Rectal mucus and blood	1 High CRP 1 High D-dimer 1 Anemia 1 Low Hb	2 Acute ISN	0	0	0	2 Surgical reductions of the ISN 1 Rockey–Davis incision for manual reduction 1 Anticoagulant No failure (n = 2) No recurrence (n = 2)	(Modified NOS, high) 2 Survived
Moazzam et al., 2020 [29], Pakistan	Retrospective case report, single center	4	1 (100)	1 Pakistani	1 No medical history	1	1	1 Ileocolic ISN AND 1 Abdominal pain 1 Crying 1 Drawing up of the legs towards the abdomen 1 Anorexia 1 Red currant jelly stools 1 Pallor 1 Irritability 1 Sausage-shaped lump 1 Anemia	1 High D-dimer 1 Low Hb	1 Acute ISN	0	0	0	1 Pneumatic reduction of the ISN 1 Antibiotics 1 Analgesics No failure (n = 1) No recurrence (n = 1)	(Modified NOS, high) 1 Survived
Noviello et al., 2021 [30], Italy	Retrospective case report, single center	7	1 (100)	1 White (Caucasian)	1 No medical history	1	1	1 Ileocolic ISN AND 1 Abdominal pain 1 Anorexia 1 Diarrhea 1 Vomiting 1 Sleepiness 1 Pallor 1 Lethargy 1 Dehydration 1 Red currant jelly stools	1 High D-dimer	1 Acute ISN 1 Alternating rings of low and high echogenicity 1 “Pseudokidney” sign	1	0	0	1 Hydrostatic reduction of the ISN 1 Laparotomy 1 Surgical resection 1 Anastomosis Yes failure (hydrostatic, n = 1) No recurrence (n = 1)	(Modified NOS, high) 1 Survived
Osorno et al., 2021 [31], Colombia	Retrospective case report, single center	8	1 (100)	1 Hispanic	1 No medical history	1	1	1 Ileocolic ISN AND 1 Abdominal pain 1 Red currant jelly stools 1 Perception of a firm and painful mass 1 Metabolic acidosis 1 Peritonitis	1 Not reported	1 Acute ISN 1 Ischemia of distal ileum and right colon	1	0	0	1 Laparotomy 1 Surgical resection 1 Ileostomy 1 Fistula No failure (n = 1) No recurrence (n = 1)	(Modified NOS, high) 1 Survived
Ponmani and Hulse 2022 [32], United Kingdom	Retrospective case report, single center	18	0 (0)	1 White (Caucasian)	1 No medical history	1	1	1 Ileocolic ISN AND 1 Constipation 1 Abdominal pain	1 High CRP	1 Acute ISN 1 Doughnut-shaped mass	0	0	0	1 Pneumatic reduction of the ISN No failure (n = 1) No recurrence (n = 1)	(Modified NOS, high) 1 survived
Rajalakshmi et al., 2020 [7], India	Retrospective case report, single center	8	1 (100)	1 Indian	1 No medical history	1	1	1 Ileocolic ISN AND 1 Red currant jelly stools 1 Vomiting 1 Lethargy 1 Dehydration 1 Perception of a firm and painful mass	1 Low Hb 1 Low hematocrit	1 Acute ISN	0	0	0	1 IV fluids 1 Pneumatic reduction of the ISN No failure (n = 1) No recurrence (n = 1)	(Modified NOS, high) 1 Survived
Rohani et al., 2021 [33], Iran	Retrospective case report, single center	72	1 (100)	1 Persian	1 No medical history	1	1	1 Location was not reported AND 1 Abdominal tenderness 1 Abdominal pain 1 Vomiting 1 Diarrhea 1 Abdominal distension	1 Leukopenia 1 Decreased lymphocytes 1 High calprotectin 1 Hypoalbuminemia 1 Hypocalcemia 1 Hypophosphatemia 1 Hypomagnesaemia 1 Raised liver enzymes	1 Multifocal small bowel loops intussusceptum 1 Pneumatosis intestinalis in ascending colon 1 Dilatation in colon caliber 1 Necrotizing enterocolitis	0	0	0	1 Oral rehydration solution 1 Ondansetron 1 Total parenteral nutrition 1 Antibiotics No failure (n = 1) No recurrence (n = 1)	(Modified NOS, high) 1 Survived
Salman et al., 2022 [34], United States	Retrospective cohort, single center	Median (IQR), 36 (3-216)	19 (79.2)	4 Whites (Caucasians) 6 Hispanics 1 Asian	11 Not reported	685	24	8 Small bowel ISN and 3 ileocolic ISN AND 7 Vomiting 5 Abdominal pain 2 Blood in stool 4 Red currant jelly stools	11 Not reported	11 Abnormal imaging 3 Acute ISN	11 Not reported	11 Not reported	11 Not reported	3 Pneumatic reductions of the ISN 4 Surgical reductions of the ISN No failure (n = 8) Yes failure (pneumatic, n = 3) No recurrence (n = 11)	(NOS, 7) 11 Survived
Saxena et al., 2021 [35], United Kingdom	Retrospective case report, single center	10	0 (0)	1 White (Caucasian)	1 No medical history	1	1	1 Location was not reported AND 1 Red currant jelly stools 1 Vomiting	1 Not reported	1 Acute ISN 1 Waugh syndrome (ISN with malrotation)	0	0	0	1 Pneumatic reduction of the ISN 1 Surgical reduction of the ISN 1 Ladd’s procedure Yes failure (pneumatic, n = 1) No recurrence (n = 1)	(Modified NOS, high) 1 Survived
Scottoni et al., 2022 [36], Italy	Retrospective case reports, multicenter	1 and 5.5	1 (50)	2 Whites (Caucasians)	1 No medical history 1 Hyperinsulinism	2	2	2 Ileocolic ISN AND 1 Constipation 2 Vomiting 2 Lethargy 1 Dehydration 2 Blood in stool 1 Anorexia	2 Not reported	2 Acute ISN 1 Enlarged lymph nodes 1 Necrosis of the terminal ileum to the splenic flexure	1	1	1	1 Pneumatic reduction of the ISN 1 Laparotomy 1 Removal of lymph node 1 Hemicolectomy 1 Anastomosis 1 Noradrenaline No failure (n = 1) Yes failure (pneumatic, n = 1) No recurrence (n = 2)	(Modified NOS, high) 2 Survived
Sorkhi et al., 2022 [37], Iran	Retrospective case report, single center	132	1 (100)	1 Persian	1 Nephrotic syndrome 1 Long use of steroid (15 years)	1	1	1 Ileocolic ISN AND 1 Abdominal pain 1 Vomiting 1 Nausea 1 Weakness 1 Anorexia 1 Myalgia 1 Pallor 1 Dehydration	1 High leukocytes 1 Hypernatremia 1 Hypokalemia	1 Acute ISN 1 Meckel’s diverticulum	0	0	0	1 Steroids 1 Enalapril 1 Pantoprazole 1 Antibiotics 1 IV fluids 1 Hydrostatic reduction of the ISN 1 Diverticulectomy No failure (n = 1) No recurrence (n = 1)	(Modified NOS, moderate) 1 Survived
Stephenson et al., 2021 [38], United States	Retrospective case report, single center	6	1 (100)	1 White (Caucasian)	1 No medical history	1	1	1 Ileocolic ISN AND 1 Abdominal pain 1 Vomiting 1 Irritability 1 Red currant jelly stools	1 Not reported	1 Acute ISN	0	0	0	1 Pneumatic reduction of the ISN Yes failure (pneumatic, n = 1) Yes recurrence (n = 1)	(Modified NOS, high) 1 Survived
Sullivan et al., 2021 [39], United States	Retrospective case report, single center	7	1 (100)	1 White (Caucasian)	1 No medical history	1	1	1 Ileal-ileal ISN and 1 ileocolic ISN AND 1 Abdominal pain 1 Crying 1 Fist clenching 1 Grimacing	1 Not reported	1 Acute ISN 1 Ileocolic ISN (Target sign)	0	0	0	1 Hydrostatic reduction of the ISN Yes failure (hydrostatic, n = 1) Yes recurrence (n = 1)	(Modified NOS, high) 1 Survived
Swyden et al., 2022 [40], United States	Retrospective case report, single center	4	1 (100)	1 White (Caucasian)	1 No medical history	1	1	1 Ileocolic ISN AND 1 Blood in stool 1 Anorexia 1 Red currant jelly stools 1 Tachycardia 1 Lethargy 1 Metabolic acidosis	1 Thrombocytosis 1 High leukocytes 1 Hypochloremia 1 Raised procalcitonin	1 Acute ISN 1 Doughnut-shaped mass 1 Pneumoperitoneum	0	0	0	1 IV fluids 1 Hydrostatic reduction of the ISN 1 Laparotomy Yes failure (hydrostatic, n = 1) No recurrence (n = 1)	(Modified NOS, high) 1 Survived
Tran et al., 2022 [41], United States	Retrospective case report, single center	8	0 (0)	1 White (Caucasian)	1 No medical history	1	1	1 Location was not reported AND 1 Blood in stool 1 Rash 1 Diarrhea 1 Metabolic acidosis	1 High leukocytes 1 High CRP	1 Acute ISN (target sign)	0	0	0	1 No treatment for ISN (follow-up and bowel rest) No failure (n = 1) No recurrence (n = 1)	(Modified NOS, moderate) 1 Survived
Vecchio et al., 2021 [42], Italy	Retrospective cohort, multicenter	4 Not reported	4 Not reported	4 Whites (Caucasians)	4 Not reported	685	4	1 Ileal ISN and 3 ileocolic ISN AND 4 Abdominal pain 4 Vomiting 4 Red currant jelly stools	4 Not reported	4 Not reported	4 Not reported	4 Not reported	4 Not reported	2 Surgical reductions of the ISN 1 Removal of solid mass Failure was not reported (n = 4) Recurrence was not reported (n = 4)	(NOS, 8) 4 Not reported
Wang et al., 2021 [43], China	Retrospective cohort, single center	10	0 (0)	1 Asian	1 No medical history	238	1	1 Location was not reported AND 1 Blood in stool 1 Vomiting 1 Diarrhea 1 Oliguria 1 Acute kidney injury (stage 3)	1 Raised procalcitonin 1 High CRP 1 High interleukin-6 1 High D-dimer 1 High LDH 1 High BUN 1 High serum creatinine 1 Low estimated glomerular filtration rate 1 Proteinuria 1 Hematuria	1 Acute ISN 1 Intestinal necrosis	1	1	1	1 Surgical reduction of the ISN 1 Antibiotics 1 Antivirals 1 Steroids 1 IVIG 1 CRRT 1 Plasma exchange Yes failure (surgical, n = 1) No recurrence (n = 1)	(NOS, 7) 1 Died
Yalçınkaya et al., 2021 [44], Turkey	Retrospective cohort, single center	72	0 (0)	1 White (Caucasian)	1 No medical history	1	1	1 Ileo-ileal ISN AND 1 Diarrhea 1 Abdominal tenderness 1 Abdominal pain 1 Conjunctivitis 1 Tachycardia 1 Abdominal tenderness 1 Blood in stool 1 Mitral regurgitation 1 Left ventricular systolic dysfunction	1 High CRP 1 Raised ESR 1 Raised procalcitonin 1 High interleukin-6 1 High ferritin 1 High fibrinogen 1 Decreased lymphocytes	1 Acute ISN 1 Fluid retention in the ileal and colonic walls	0	0	0	1 IVIG 1 Steroids 1 No treatment for ISN (follow-up and bowel rest) No failure (n = 1) No recurrence (n = 1)	(NOS, 7) 1 Survived

APTT, activated partial thromboplastin time; ARDs, acute respiratory distress syndrome; BP, blood pressure; BUN, blood urea nitrogen; COVID-19, coronavirus disease 2019; CRRT, continuous renal replacement therapy; CRP, C-reactive protein; DIC, disseminated intravascular coagulation; ESR, erythrocyte sedimentation rate; Hb, hemoglobin; ICU, intensive care unit; ISN, intussusception; IQR, interquartile range; IV, intravenous; IVIG, IV immunoglobulin; LDH, lactate dehydrogenase; NGT, nasogastric tube; NOS, Newcastle-Ottawa Scale; NT-proBNP, N-terminal pro b-type natriuretic peptide; SARS-CoV-2, severe acute respiratory syndrome coronavirus 2; WBCs, white blood cells; WHO, World Health Organization. ^a^ Data are presented as the median (25th–75th percentiles).

**Table 2:** Demographic data of the pediatric SARS-CoV-2 patients with ISN, stratified by treatment outcome (n = 34 studies), 2020–2022.

Variable	Findings ^a^			
	All (n = 64)	Survived (n = 43)	Died (n = 4)	*p*-Value ^b^
Age				
Less than 1 month	0	0	0	0.001 *
1 month to less than 1 year	32 (50)	28 (65.1)	4 (100)	
1 year to less than 3 years	4 (6.2)	4 (9.3)	0	
3 years to less than 6 years	4 (6.2)	4 (9.3)	0	
6 years to less than 12 years	7 (10.9)	7 (16.3)	0	
12 years to 18 years	0	0	0	
Gender				
Male	41 (64.1)	41 (95.3)	0	0.000 *
Female	18 (28.1)	14 (32.5)	4 (100)	
Ethnicity				
White (Caucasian)	25 (39.1)	25 (58.1)	0	0.000 *
Hispanic	13 (20.3)	13 (30.2)	0	
Asian	5 (7.8)	1 (2.3)	4 (100)	
Persian	2 (3.1)	2 (4.6)	0	
Indian	3 (4.7)	3 (7)	0	
Arab	1 (1.6)	1 (2.3)	0	
Pakistani	1 (1.6)	1 (2.3)	0	
ISN classification by location				
Ileocolic	34 (53.1)	32 (74.4)	2 (50)	0.001 *
Ileo-ileal	4 (6.2)	4 (9.3)	0	
Location was not reported	5 (7.8)	3 (7)	2 (50)	
Imaging				
Acute ISN	37 (57.8)	33 (76.7)	4 (100)	0.237
Enlarged lymph nodes	6 (9.4)	6 (13.9)	0	
Target sign	4 (6.2)	4 (9.3)	0	
Imaging findings were not reported	4 (6.2)	4 (9.3)	0	
Intestinal necrosis	4 (6.2)	2 (4.6)	2 (50)	
Doughnut-shaped mass	2 (3.1)	2 (4.6)	0	
Meckel’s diverticulum	2 (3.1)	2 (4.6)	0	
Symptoms from ISN				
Vomiting	36 (56.2)	32 (74.4)	4 (100)	0.179
Abdominal pain	29 (45.3)	29 (67.4)	0	
Red currant jelly stools	25 (39.1)	23 (53.5)	2 (50)	
Blood in stool	15 (23.4)	13 (30.2)	2 (50)	
Anorexia	9 (14.1)	9 (20.9)	0	
Irritability	8 (12.5)	8 (18.6)	0	
Abdominal tenderness	8 (12.5)	8 (18.6)	0	
Dehydration	7 (10.9)	7 (16.3)	0	
Pallor	6 (9.4)	6 (13.9)	0	
Crying	8 (12.5)	6 (13.9)	2 (50)	
Lethargy	7 (10.9)	6 (13.9)	1 (25)	
Abdominal distension	7 (10.9)	5 (11.6)	2 (50)	
Diarrhea	6 (9.4)	5 (11.6)	1 (25)	
Tachycardia	4 (6.2)	4 (9.3)	0	
Perception of a firm and painful mass	4 (6.2)	4 (9.3)	0	
Rectal mucus and blood	3 (4.7)	3 (7)	0	
Metabolic acidosis	3 (4.7)	3 (7)	0	
Low BP	2 (3.1)	2 (4.6)	0	
Anemia	2 (3.1)	2 (4.6)	0	
Weakness	2 (3.1)	2 (4.6)	0	
Nausea	3 (4.7)	2 (4.6)	1 (25)	
Cold extremities	2 (3.1)	2 (4.6)	0	
Constipation	2 (3.1)	2 (4.6)	0	
DIC	2 (3.1)	0	2 (50)	
Septic shock	2 (3.1)	0	2 (50)	
Multiple organ dysfunction	2 (3.1)	0	2 (50)	
Peritonitis	2 (3.1)	1 (2.3)	1 (25)	
Drowsiness	2 (3.1)	1 (2.3)	1 (25)	
Absence of bowel sounds	2 (3.1)	1 (2.3)	1 (25)	
Laboratory findings				
Laboratory results were not reported	25 (39.1)	25 (58.1)	0	0.063
High CRP	14 (21.9)	10 (23.2)	4 (100)	
High D-dimer	9 (14.1)	7 (16.3)	2 (50)	
Low Hb	6 (9.4)	6 (13.9)	0	
Raised procalcitonin	5 (7.8)	3 (7)	2 (50)	
High leukocytes	4 (6.2)	4 (9.3)	0	
Decreased lymphocytes	4 (6.2)	3 (7)	1 (25)	
High ferritin	4 (6.2)	3 (7)	1 (25)	
High LDH	4 (6.2)	2 (4.6)	2 (50)	
High WBCs	3 (4.7)	3 (7)	0	
High interleukin-6	3 (4.7)	1 (2.3)	2 (50)	
High neutrophils	2 (3.1)	2 (4.6)	0	
Raised liver enzymes	2 (3.1)	2 (4.6)	0	
Hypokalemia	2 (3.1)	2 (4.6)	0	
High BUN	2 (3.1)	0	2 (50)	
Proteinuria	2 (3.1)	1 (2.3)	1 (25)	
High prothrombin time	2 (3.1)	1 (2.3)	1 (25)	
Leukopenia	2 (3.1)	1 (2.3)	1 (25)	
Hypoalbuminemia	2 (3.1)	1 (2.3)	1 (25)	
Hypocalcemia	2 (3.1)	1 (2.3)	1 (25)	
Comorbidities				
No medical history	32 (50)	28 (65.1)	4 (100)	0.036 *
Not reported	15 (23.4)	15 (34.9)	0	
Left-sided renal agenesis	1 (1.5)	1 (2.3)	0	
Developmental delay	1 (1.5)	1 (2.3)	0	
Meningism	1 (1.5)	1 (2.3)	0	
Hyperinsulinism	1 (1.5)	1 (2.3)	0	
Nephrotic syndrome	1 (1.5)	1 (2.3)	0	
Long use of steroid (15 years)	1 (1.5)	1 (2.3)	0	
Treatment				
Surgical reduction of the ISN	17 (26.6)	15 (34.9)	2 (50)	0.051
Pneumatic reduction of the ISN	13 (20.2)	11 (25.6)	2 (50)	
Antibiotics	12 (18.7)	8 (18.6)	4 (100)	
Hydrostatic reduction of the ISN	11 (17.2)	11 (25.6)	0	
Laparotomy	10 (15.6)	9 (20.9)	1 (25)	
IV fluids	8 (12.5)	8 (18.6)	0	
Oxygen supplementation	6 (9.4)	4 (9.3)	2 (50)	
Analgesics	5 (7.8)	5 (11.6)	0	
Surgical resection	5 (7.8)	4 (9.3)	1 (25)	
Steroids	5 (7.8)	3 (7)	2 (50)	
Anastomosis	3 (4.7)	3 (7)	0	
Anticoagulant	2 (3.1)	2 (4.6)	0	
Total parenteral nutrition	2 (3.1)	2 (4.6)	0	
No. treatment for ISN (follow-up and bowel rest)	2 (3.1)	2 (4.6)	0	
CRRT	2 (3.1)	0	2 (50)	
Plasma exchange	2 (3.1)	0	2 (50)	
Interferon	2 (3.1)	0	2 (50)	
Antivirals	2 (3.1)	0	2 (50)	
IVIG	3 (4.7)	1 (2.3)	2 (50)	
Ladd’s procedure	2 (3.1)	1 (2.3)	1 (25)	
Inotropes	2 (3.1)	1 (2.3)	1 (25)	
Dopamine	2 (3.1)	1 (2.3)	1 (25)	
ISN was recurrent				
No	45 (70.3)	41 (95.3)	4 (100)	0.140
Yes	2 (3.1)	2 (4.6)	0	
Failure to ISN treatment				
No failure	25 (39.1)	23 (53.5)	2 (50)	0.002 *
Pneumatic reduction (yes)	7 (10.9)	6 (13.9)	1 (25)	
Hydrostatic reduction (yes)	6 (9.4)	6 (13.9)	0	
Surgical reduction (yes)	1 (1.5)	0	1 (25)	
Complications and treatment outcomes				
Patient was admitted to ICU	9 (14.1)	5 (11.6)	4 (100)	0.000 *
Patient was intubated and on mechanical ventilation during the ICU stay	6 (9.4)	2 (4.6)	4 (100)	0.000 *
Patient experienced ARDS	6 (9.4)	2 (4.6)	4 (100)	0.000 *

ARDS, acute respiratory distress syndrome; BP, blood pressure; BUN, blood urea nitrogen; COVID-19, coronavirus disease 2019; CRRT, continuous renal replacement therapy; CRP, C-reactive protein; DIC, disseminated intravascular coagulation; GFR, glomerular filtration rate; Hb, hemoglobin; ICU, intensive care unit; ISN, intussusception; IV, intravenous; IVIG, IV immunoglobulin; LDH, lactate dehydrogenase; SARS-CoV-2, severe acute respiratory syndrome coronavirus 2; WBCs, white blood cells. ^a^ Data are presented as the number (%). ^b^ Chi-square (*χ*^2^) test was used to compare between the survival and death groups. Percentages do not total 100% owing to missing data. * Represents significant differences.

**Table 3:** Predictors of mortality in pediatric patients hospitalized for ISN and SARS-CoV-2 (n = 64).

Variable	Univariate Analysis OR (95% CI) for Death		Multivariate Analysis OR (95% CI) for Death	
	OR (95% CI)	*p*-Value	OR (95% CI)	*p*-Value
Age (1 month to <1 year)	0.42 (0.1–0.33)	0.04 *	0.1 (0.13–0.25)	0.52
Gender (Female)	0.74 (0.36–0.52)	<0.001 *	1.13 (0.31–0.79)	0.045 *
Location (Asia)	0.36 (0.26–0.45)	<0.001 *	0.38 (0.28–0.48)	<0.001 *
ISN symptom (abdominal pain)	0.14 (0.43–0.71)	0.62	0.19 (0.13–0.3)	0.41
ISN symptom (blood in stool)	0.14 (0.41–0.7)	0.61	0.32 (0.11–0.31)	0.34
ISN symptom (constipation)	0.5 (0.15–1.15)	0.13	0.7 (0.22–0.45)	0.36
ISN symptom (crying)	0.13 (0.44–0.69)	0.66	NA	NA
ISN symptom (diarrhea)	0.17 (0.41–0.74)	0.57	NA	NA
ISN symptom (DIC)	1 (0.35–1.65)	0.003 *	1.31 (0.47–0.97)	0.42
ISN symptom (drowsiness)	0.5 (0.21–1.15)	0.13	0.68 (0.2–0.41)	0.33
ISN symptom (lethargy)	0.33 (0.24–0.91)	0.25	NA	NA
ISN symptom (multiple organ failure)	1 (0.35–1.65)	0.003 *	1.31 (0.47–0.97)	0.42
ISN symptom (nausea)	0.33 (0.28–0.95)	0.29	0.41 (0.22–0.47)	0.43
ISN symptom (peritonitis)	0.5 (0.15–1.15)	0.13	0.7 (0.22–0.45)	0.36
ISN symptom (red currant jelly stools)	0.04 (0.5–0.58)	0.88	0.32 (0.51–0.87)	0.32
ISN symptom (vomiting)	0.11 (0.43–0.66)	0.68	0.39 (0.24–0.68)	0.75
Laboratory finding (decreased lymphocytes)	0.25 (0.77–1.27)	0.62	1.01 (0.74–0.75)	0.22
Laboratory finding (high bloods urea nitrogen)	1 (0.12–2.12)	0.08	0.86 (0.33–0.56)	0.48
Laboratory finding (high C reactive protein)	0.31 (0.64–1.25)	0.52	0.71 (0.2–0.68)	0.47
Laboratory finding (high D-dimer)	0.22 (0.74–1.18)	0.64	0.48 (0.24–0.71)	0.55
Laboratory finding (high ferritin)	0.25 (0.77–1.27)	0.62	0.38 (0.27–0.4)	0.48
Laboratory finding (high interleukin-6)	0.67 (0.38–1.72)	0.21	0.6 (0.38–0.74)	0.64
Laboratory finding (high lactate dehydrogenase)	0.5 (0.52–1.52)	0.33	NA	NA
Laboratory finding (high prothrombin time)	0.5 (0.62–1.62)	0.37	NA	NA
Laboratory finding (hypocalcemia)	0.5 (0.62–1.62)	0.37	0.21 (0.1–0.35)	0.49
Laboratory finding (leukopenia)	0.5 (0.62–1.62)	0.37	NA	NA
Laboratory finding (proteinuria)	0.5 (0.62–1.62)	0.37	NA	NA
Failure to ISN reduction (pneumatic or surgical) (yes)	0.16 (0.06–0.27)	0.002 *	0.11 (0.05–0.21)	0.036 *
Recurrent ISN (yes)	0.17 (0.09–0.43)	0.21	0.34 (0.19–0.29)	0.51
Intensive care unit admission (yes)	0.53 (0.45–0.6)	<0.001 *	0.71 (0.83–1.18)	0.03 *
Mechanically ventilated (yes)	0.84 (0.79–0.9)	<0.001 *	0.68 (0.51–1.41)	0.01 *
Suffered from ARDS (yes)	0.75 (0.68–0.81)	<0.001 *	0.88 (0.93–1.88)	0.01 *
Treatment (antibiotics = yes)	0.31 (0.02–0.59)	0.03 *	0.29 (0.01–0.03)	0.06
Treatment (antivirals = yes)	1 (0.61–1.39)	<0.001 *	0.78 (0.93–1.22)	0.77
Treatment (CRRT = yes)	1 (0.55–1.45)	<0.001 *	0.64 (0.31–0.45)	0.63
Treatment (dopamine = yes)	0.5 (0.05–0.95)	0.03 *	NA	NA
Treatment (inotropes = yes)	0.5 (0.05–0.95)	0.03 *	NA	NA
Treatment (interferon = yes)	1 (0.55–1.45)	<0.001 *	0.64 (0.31–0.45)	0.63
Treatment (IVIG = yes)	0.75 (0.39–1.11)	<0.001 *	0.78 (0.48–1.49)	0.27
Treatment (Ladd’s procedure = yes)	0.5 (0.05–0.95)	0.03 *	NA	NA
Treatment (oxygen supplementation = yes)	0.33 (0.61–1.39)	0.04 *	0.19 (0.17–0.34)	0.23
Treatment (plasma exchange = yes)	1 (0.55–1.45)	<0.001 *	0.64 (0.31–0.45)	0.63
Treatment (pneumatic reduction of ISN = yes)	0.03 (0.23–0.28)	0.83	0.13 (0.16–0.45)	0.61
Treatment (surgical reduction of ISN = yes)	0.09 (0.16–0.34)	0.47	NA	NA
Treatment (steroids = yes)	0.5 (0.17–0.83)	<0.001 *	0.21 (0.14–0.58)	0.21

ARDS, acute respiratory distress syndrome; CRRT, continuous renal replacement therapy; DIC, disseminated intravascular coagulation; ISN, intussusception; IVIG, intravenous immunoglobulin; NA, not applicable. * Represents significant differences.

## Data Availability

Data are available upon request; please contact the author for data requests.

## Abbreviations

COVID-19, coronavirus disease 2019; NOS, Newcastle-Ottawa scale; PRISMA, Preferred Reporting Items for Systematic Reviews and Meta-Analyses; SARS-CoV-2, severe acute respiratory syndrome coronavirus 2; ISN, intussusception; DIC, disseminated intravascular coagulation; ARDS, acute respiratory distress syndrome; CRRT, continuous renal replacement therapy; LDH, lactate dehydrogenase; IVIG, intravenous immunoglobulin.
